# Preference and Practice of Traditional Medicine and Associated Factors in Jimma Town, Southwest Ethiopia

**DOI:** 10.1155/2021/9962892

**Published:** 2021-05-29

**Authors:** Belachew Umeta Chali, Abush Hasho, Nimona Berhanu Koricha

**Affiliations:** School of Pharmacy, Institute of Health, Jimma University, Jimma, Oromia, Ethiopia

## Abstract

**Background:**

Traditional medicine is the sum total of knowledge, talents, and practices that are used to uphold health, as well as to avert, identify, improve, or treat illnesses. Sociodemographic/economic characteristics, culture, and environment can influence the preference and practice of traditional medicine.

**Objective:**

To assess the preference and practices of traditional medicine and associated factors.

**Methods:**

A community-based cross-sectional study was conducted among 271 residents of Jimma town. The data were collected by interviewing selected households. The households were selected by a simple random sampling technique. The data were analysed using SPSS version 20. Descriptive statistics were used for organizing, describing, and summarizing the data. Chi-square (*X*^2^) test was used to identify factors associated with the preference and practice of traditional medicine.

**Results:**

More than half (221(81.5%)) of the participants practiced traditional medicine. Religion (*X*^2^ = 17.18; *p*=0.001), marital status (*X*^2^ = 15.42; *p*=0.001), occupation (*X*^2^ = 19.74; *p*=0.001), and educational level (*X*^2^ = 28.39; *p* ≤ 0.001) were the sociodemographic factors determining the use of traditional medicine. However, 25 (9.2%) of the participants preferred to use traditional medicine. Affordability (25 (100%)), religious affiliation (21 (84%)), and distance from home (20 (80%)) were some reasons for preference. Educational level (*X*^2^ = 15.56; *p*=0.04), marital status (*X*^2^ = 12.39; *p*=0.04), and occupation (*X*^2^ = 15.61; *p*=0.003) were the factors affecting their preference for traditional medicine.

**Conclusion:**

A majority of the participants practiced traditional medicine use. Religion, marital status, occupation, and educational level were factors affecting the practice of the participants. More than half of the participants did not prefer to use traditional medicine. Affordability, religious affiliation, and distance from home were some reasons for preferring traditional medicine.

## 1. Introduction

Traditional medicine, an art of treatment practices, strategies, knowledge, and beliefs including plant-, animal-, and mineral-based medicines, spiritual therapies, manual techniques, and workouts applied singularly or in combination to treat, identify, and prevent illnesses or uphold the well-being. Since ancient times, humans have been using natural products, such as plants, animals, microorganisms, and oceanic organisms, in remedies to prevent or treat illnesses [1, 2]. The recognition of these practices as standard healthcare possibilities will have an incredible influence in the cost of healthcare interventions, protective medicine, and self-healing [3, 4].

As to the WHO, 65–80% of the world's healthcare practice includes the use of traditional medicine in some way [5]. There has been a continuing demand for and popular use of traditional medicine worldwide [6]. In some lower- and middle-income countries, traditional healers remain the only or main health providers for millions of people living in rural areas. For instance, the ratio of traditional health experts to the population in Africa is 1 : 500, while the ratio of physicians to the population is 1 : 40,000 [7].

In Africa, traditional medicine is part of the first set of response mechanisms for medical emergencies, whereas in others, the whole health system of the community relies on medicines embedded in indigenous practice and belief [8]. This is due to the fact that modern pharmaceuticals and medical procedures remain unreachable to a large number of African people due to their relatively high cost and concentration of health facilities in urban centers [9, 10].

Despite the high consideration given to the traditional medicine practice around the world, it seems to face noteworthy challenges [3]. The most important recognized challenge is the lack of a reference standard for determining the proper dosage of the traditional medicine for the patients. This in turn has resulted in the creation of incorrect and incomplete information about the traditional medicine drugs. Another very important challenge is the lack of national policy to manage and legalize the practice of using traditional medicine [11].

In the Ethiopian context, there seems to be no village, town, or city where traditional medicine is not involved in the delivery of healthcare since it is an integral part of the local culture and accessible to the majority of the population [12]. Contrary to its wide usage, traditional medicine in Ethiopia is not uniformly practiced. The users are considerably diverse and significantly vary between regions [13]. The standard policies and regulations governing traditional medicine are too weak. Instead, the users are associated with cultural beliefs. Subsequently, traditional medicine is an integral part of a community's identity and value in that it is difficult to regulate through a nationwide framework concerning its safety and effectiveness using different medicine categorizations and descriptions [14, 15].

With regard to determinants of traditional medicine practice in Ethiopia, some literature studies hypothetically linked the affordability of traditional medicine to the fact of its use by up to 80% of the population, while others added deprived access to modern healthcare facilities [16–18]. Cultural belief, monthly income, and religious belief have also been mentioned as determinants [17, 19]. Yet, there is still scarcity of information about the common preferable traditional medicine practices and reasons contributing to the link between traditional medicine practice and sociodemographic, economic, or individual difference and environmental factors.

Therefore, it is appropriate to assess the preference and practice of traditional medicine and its determinants and reasons for preferring the practices and possible factors behind the common traditional medicine being practiced among the community. So, the current study aimed to assess the preference and practice of traditional medicine and associated factors. The result of the study can be used as an input for policy makers and other stakeholders to identify and prepare directives to control and legalize the traditional medicine practices.

## 2. Materials and Methods

### 2.1. Study Area and Period

The study was conducted in Jimma town. The town is located at 352 km southwest of Addis Ababa, the capital city of Ethiopia. According to the Central Statistical Agency (CSA, 2007) report, the total population of the town was 120,960, of whom 60,824 were males. A total of 32,191 households were present in the town [20]. According to the data from the Jimma Zone Health Bureau, there are five public health centers, one medical center, and two general hospitals. The study was conducted from November to December 2020.

### 2.2. Study Design

It is community-based cross-sectional study design.

### 2.3. Participants and Sampling Procedure

The sample size was determined using the simple population proportion formula for the cross-sectional study, *n* = *Z*^2^*P* (1−*P*)/*d*^2^, where *P* = 80% prevalence of traditional medicine use [18] and *Z* = 1.96 is the value under the standard normal table for the confidence level of 95%, margin of error 5%, and nonresponse rate of 10%. Finally, 271 households were used as a sample size, and adults older than 18 years and lived for at least 6 months were included in the study. Parents who are unable to give the required information due to any reason were excluded from the study. Priority was given for mothers and fathers, and if they are unavailable during data collection, any individual older than 18 years was included.

### 2.4. Data Collection Tools and Techniques

A pretested, structured questionnaire was adopted from a previously conducted study with some modifications based on the local context [18, 19]. The pretest was conducted on 5% nonsampled respondents to check the validity, reliability, and clarity of the questionnaire. The questionnaire was prepared in English. It consists of the following parts: baseline information (age, sex, ethnicity, religion, marital status, occupation, educational level, and monthly income), the practice of traditional medicine (ever heard of traditional medicine before, current use of TM, frequency of use, behavior of users to inform their physicians, safe to mix TM with modern medicine, common types of traditional medicine used, by whom traditional medicine was practiced, and from whom information was obtained about traditional medicine use), and preference (preference between traditional and modern medicine and reason for the preference of traditional medicine). Data were collected using structured interview questionnaires. The list of all households in the town was obtained from the Jimma town Administration Office. The list was arranged in the ascending order according to the registration number given for each house by the town administration. Finally, a simple random sampling technique was used to select the required quantity of households. Trained graduating pharmacy students collected the data.

### 2.5. Data Processing and Analysis

The data were entered and cleaned by using Epi Info software and exported to SPSS version 20 for further analysis. Descriptive statistics were used for organizing, describing, and summarizing the data. Chi-square (*X*^2^) was used to check the association of independent variables with the dependent variable. *P* value <0.05 was used to declare the significant association.

### 2.6. Ethical Consideration

The study protocol was reviewed and approved by the Ethical Review Committee of Jimma University (JU). The Ethical Review Committee of Jimma University accepted and approved verbal informed consent. Verbal informed consent was obtained from the participants after the purpose and methods of the study had been explained in detail, and all of their responses were kept confidential and anonymous.

## 3. Results

### 3.1. Sociodemographic Characteristics

A majority of the respondents were females and married. More than half of the respondents were Muslims. More than half of the participants had a monthly income of <2000 birr which is about 50.1 USD (Table 1).

### 3.2. Practice of Traditional Medicine

The prevalence of TM practice was 211 (81.5%). Of those who practiced traditional medicine, 160 (72.4%) of them informed their physician about the traditional medicine they used. 184 (83.3%) of them reported that they only have symptomatic relief after the use of traditional medicine. More than half (120 (54.3%)) of the respondents obtained information on the use, benefit, and efficacy of traditional medicine from their family members. The common ailments for which traditional medicine was used were cough, headache, and intestinal/stomach infections. The majority of the participants reported that medical herbs (118 (54.4%)) are the commonly used traditional medicine type. Most (137 (62%)) of the participants did not check the safety of traditional medicine when they used it in combination with a prescription drug, and 141 (63.8%) of the participants obtained traditional medicine they used from traditional medicine healer (Table 2).

### 3.3. Preference of the Community for the Management of Different Ailments for the Future

The majority (204 (75.3%)) of them preferred modern medicine, and only 25 (9.2%) of the participants preferred the use of traditional medicine for any types of ailments. The reasons for the preference of traditional medicine were affordability (25 (100.0%)) and religious affiliation (21 (84.0%)) (Table 3).

### 3.4. Factors Affecting the Practice and Preference of Traditional Medicine

Religion, marital status, occupation, and educational level were the factors affecting the practice of traditional medicine in the study area. Marital status, occupation, and educational level were the sociodemographic characteristics affecting their preference (Table 4).

## 4. Discussion

In this study, the practice of traditional medicine was 81.5%. The finding was higher than the study conducted in North Central Ethiopia [21] which states that the practice of traditional medicine use is 35.8%. However, the practice is lower than the study conducted in Burka Jato Kebele, West Ethiopia [22], in which 92.22% of the participants rely on traditional medicine. The discrepancy might be due to the sample size difference and accessibility of health facilities. And it is almost comparable with the study conducted in Shoba Bultum, Southwest Ethiopia [19], in which 79.47% of the participants practice traditional medicine use. From those participants who used traditional medicines, 72.4% of the participants reported to their physicians that they used traditional medicine, and 83.4% of them felt that traditional medicine use for the management of different ailments only gives symptomatic relief.

Around fifty-four percent of the participants had information about the use of traditional medicine for different ailments from their family, and the most common traditional medicine used was medical herbs (53.4%), and the finding was similar to the study conducted in North Central, West, and Southeast Ethiopia [21–23]. The most common ailments for which the community uses those traditional medicines were cold/influenza/cough, headache, and intestinal infections.

In this study, 75.3% of the participants prefer to use modern medicine for any type of ailments. The report value was smaller than the study conducted in North Central and Southeast Ethiopia [21, 23] in which 69.7% and 75.52% of the participants prefer to use traditional medicines. The dissimilarity might be due to the social, cultural, and economic difference of study areas.

Different factors affected the practice of traditional medicine in the study area, from which religion, marital status, and occupation were the factors determining the practice of traditional medicine in the study area. This might be due to community perception for some diseases to be cured by traditional medicine only [22]. The other factors affecting the practice of traditional medicine were marital status and occupation. The cost to use traditional and modern medicines is different and cheap for traditional medicine use which might be the reason for the association. The report was comparable with the study conducted in Northwest Ethiopia which indicated the relationships of occupations with knowledge, attitude, and practice of traditional medicine [18] and the study conducted in Burka Jato Kebele which reported the significance relationship between the income and practice of traditional medicine [22].

Only 9.2% of the participants preferred to use traditional medicine. The figure was different from previous studies [22, 23] which reported that 31.85% and 28.8% of the participants preferred to use traditional medicine. This difference might be due to the difference in the selection of the study site as the current study was conducted in the urban population. According to the present study, religious affiliation, therapeutic effectiveness, affordability, distance from home, and culture were reasons of the studied community to prefer traditional medicine. The finding of the study was comparable with the study conducted in Burka Jato Kebele [22]. The study conducted in Wayu, Western Ethiopia, also reported that the effectiveness of traditional medicine was the reason for preference [24]. The study also reported that there is a significant relationship between the marital status and the preference of the participants for the healthcare service in the future.

## 5. Conclusion

The majority of the participants practiced traditional medicine use. More than half of the participants did not prefer to use traditional medicine. Affordability, religious affiliation, distance from home, therapeutic effectiveness, and cultural influence were reasons for preferring traditional medicine. Marital status, occupation, religion, and educational level were determinants of the preference and practice of traditional medicine.

## 6. Limitations of the Study

Relatively small sample size, being cross-sectional in design, single site, and inclusion of only urban population were the main limitations of the study.

## Figures and Tables

**Table 1:** Sociodemographic characteristics of study participants, Jimma town, 2020.

Sociodemographic characteristics	Classification	Frequency	Percentage (%)
*Age*	18–27	95	35.1
	28–37	82	30.3
	38–47	37	13.7
	48–57	33	12.2
	>57	24	8.9

*Sex*	Male	120	44.3
	Female	151	55.7

*Ethnicity*	Oromo	218	80.4
	Amhara	31	11.4
	Gurage	6	2.2
	Tigre	2	.7
	Others^a^	14	5.2

*Religion*	Muslim	155	57.2
	Orthodox	66	24.4
	Protestant	47	17.3
	Others^b^	3	1.1

*Marital status*	Single	62	22.9
	Married	197	72.7
	Divorced	9	3.3
	Widowed	3	1.1

*Occupation*	Farmer	53	19.6
	Government worker	37	13.7
	Housewife/husband	57	21.0
	Private business owner	51	18.8
	Student	32	11.8
	Daily worker	21	7.7
	Others^c^	20	7.4

*Educational level*	Not read and write	58	21.4
	7–12	74	27.3
	Read and write	62	22.9
	>12	62	22.9
	1–6	15	5.5

*Monthly family income*	<2000	106	39.1
	2001–3800	55	20.3
	3801–6000	70	25.8
	6001–10,000	31	11.4
	>10000	9	3.3

^a^Silte, Dawro, and Yem. ^b^Waaqeffannaa and catholic. ^c^Carpenter and driver.

**Table 2:** Practice of traditional medicine in Jimma town, Southwest Ethiopia, 2020.

*Have you heard of traditional medicine?* (*n* = 271)		
	Frequency	Percentage (%)
Yes	269	99.3
No	2	0.7

*Traditional medicine practice* (*n* = 271)		
Yes	221	81.5
No	50	18.5

*Types of ailments*		
Cold/influenza/cough	133	60.2
Headache	110	49.8
Stomach/intestinal infections	86	38.9
Others^@^	34	15.4

*How frequently have you used traditional medicine?* (*n* = 221)		
Daily	1	0.5
Often	5	2.3
Sometimes	124	56.1
When unwell	91	41.2

*Behavior of users of traditional medicine to inform their physicians* (*n* = 221)		
Yes	160	72.4
No	61	27.6

*If you took traditional medicine whilst on prescription medicine, did you check it was safe to mix?* (*n* = 221)		
Yes	84	38.0
No	137	62.0

*Outcome of using traditional medicine for the management of ailments* (*n* = 221)		
Symptomatic relief	184	83.3
Permanent cure	35	15.8
No change	2	0.9

*Common types of traditional medicines used* (*n* = 221)		
Medical herbs	118	53.4
Faith healer/holy water	91	41.2
Bone setter	5	2.3
Medical herbs and holy water	3	1.4
Medical herbs, holy water, and bone setter	4	1.8

*Sources of information about traditional medicine use* (*n* = 221)		
Family	120	54.3
Friends	96	43.4
Healthcare practitioners	5	2.3

*By whom traditional medicine was practiced?* (*n* = 221)		
Traditional medicine healer	141	63.8
Elderly	10	4.5
Mother and father	70	31.7

^@^Fever, hypertension, and arteritis/joint pain.

**Table 3:** Preference of the community for traditional medicine and reason for preference, Jimma town, Southwest Ethiopia, 2020 (*N* = 271).

*Preference*	Frequency	Percentage (%)
Traditional medicine	25	9.2
Modern medicine	204	75.3
Both	42	15.5

*Reason for the preference of traditional medicine* (*N* = 25)^*∗*^		
Religious affiliations	21	84.0
Therapeutic effectiveness	20	80.0
Affordability	25	100.0
Distance from home	20	80.0
Cultural influence	14	56.0

^*∗*^The respondents respond more than one response, so the percentage might be greater than 100%.

**Table 4:** Relationships of the sociodemographic distribution of participants with the preference and practice of traditional medicine in Jimma town, Southwest Ethiopia, 2020.

Variables	Practice		Test statistics	*p* value	Preference			Test statistics	*p* value
	Yes	No			TM	MM	Both		
*Sex*									
Male	100	20	*X* ^2^ = 0.65	0.42	7	97	16	*X* ^2^ = 4.22	0.12
Female	120	31			18	107	26		

*Ethnicity*									
Oromo	182	36	*X* ^2^ = 6.62	0.16	23	157	38	*X* ^2^ = 10.38	0.24
Amhara	24	7			1	27	3		
Tigre	1	1			0	1	1		
Gurage	6	2			0	6	0		
Others^a^	7	5			1	13	0		

*Religion*									
Muslims	138	17	*X* ^2^ = 15.88	0.001	17	110	28	*X* ^2^ = 6.52	0.37
Protestants	31	16			3	39	5		
Orthodox	49	17			4	59	9		
Others^b^	2	1			1	1	0		

*Marital status*									
Married	171	26	*X* ^2^ = 14.92	0.002	21	139	37	*X* ^2^ = 12.39	0.05
Single	41	21			3	54	5		
Divorced	6	3			0	9	0		
Widowed	2	1			1	2	0		

*Occupation*									
Farmer	50	3	*X* ^2^ = 19.95	0.001	10	32	11	*X* ^2^ = 15.61	0.05
Government employee	25	12			1	32	4		
Housewife/husband	53	4			8	40	9		
Private workers	39	12			2	42	7		
Others^c^	53	20			4	58	4		

*Educational level*									
Not read and write	53	5	*X* ^2^ = 26.54	<0.001	9	34	5	*X* ^2^ = 15.56	0.04
Read and write	55	7			8	46	8		
1–6	14	1			1	12	2		
7–12	61	13			5	58	11		
>12	37	25			2	54	6		

^a^Silte, Dawro, and Yem. ^b^Waaqeffannaa and catholic. ^c^Carpenter and driver.

## Data Availability

The data used to support the findings of this study are included within the article.
